# Phytochemistry and Bioactivities of Thymol and Carvacrol: Molecular Pathways, Metabolism, and Therapeutic Insights

**DOI:** 10.1002/fsn3.71671

**Published:** 2026-04-17

**Authors:** Ahmad Mujtaba Noman, Muhammad Tauseef Sultan, Farhang Hameed Awlqadr, Hassan Raza, Shehnshah Zafar, Aimen Mazhar, Duaa Tariq, Muhammed Noori Saeed, Khaled Arab

**Affiliations:** ^1^ Department of Human Nutrition, Faculty of Food Science and Nutrition Bahauddin Zakariya University Multan Pakistan; ^2^ Food Science and Quality Control, Halabja Technical College Sulaimani Polytechnic University Sulaymaniyah Iraq; ^3^ Department of Food Science and Technology, Faculty of Food Science and Nutrition Bahauddin Zakariya University Multan Pakistan; ^4^ Department of Nutritional Analysis and Health, Kifri Technical College Garmian Polytechnic University Kifri Sulaimaniyah Iraq; ^5^ Department of Food Science and Technology, Faculty of Agriculture University of Tabriz Tabriz Iran

**Keywords:** anticancer, antimicrobial, carvacrol, food applications, thymol, *Thymus serpyllum*, toxicity

## Abstract

The concept of “One Health” has developed an inimitable bond between humans and plants, and from the dawn of humankind, plants have been consumed for both food and medicine. The use of plant‐based products has increased due to their health‐promoting potential, owing to nutritional composition and rich phytochemistry. The current review focuses on medicinal properties, safety studies, and effective applications of *Thymus serpyllum* , thymol, and carvacrol. For this, databases such as Google Scholar, PubMed, Web of Science, and ScienceDirect were utilized, along with several relevant keywords. The studies showed that *Thymus serpyllum* , a member of the genus thyme, is native to hilly regions and has been used in traditional medicine for centuries. Thymol and carvacrol are key bioactive compounds; moreover, β‐caryophyllene, γ‐terpinene, α‐thujene, linalool, gallic acid, naringin, and rutin are other phytochemicals of *T. serpyllum* , providing antioxidant, antimicrobial, anti‐inflammatory, anticancer, and antidiabetic properties. Furthermore, thymol and carvacrol are involved in immunomodulation, hepatorenal protection, cardiopulmonary protection, and the mitigation of neurodegenerative disorders. These therapeutic potentials are attributed to an improved antioxidant system, reduced IL‐6, IL‐1β, TNF‐α, NO, and modulation of signaling pathways (PI3K/Akt/mTOR/NF‐κB, Ras/MAPK). Both compounds have proved safe. However, thymol > 1000 mg/kg and carvacrol > 2480 mg/kg proved toxic in animal studies. The applications of thymol and carvacrol make them suitable choices for pharmaceutics, food technologists, veterinarians, and industrialists.

## Graphical abstract

*Thymus serpyllum* (wild thyme) contains bioactive compounds such as carvacrol, thymol, β‐caryophyllene, γ‐terpinene, and α‐thujene, along with essential vitamins and minerals. These constituents exhibit antioxidant, antimicrobial, anti‐inflammatory, and antidiabetic activities. The biological effects are associated with modulation of key signaling pathways (PI3K/Akt/mTOR, NF‐κB, and Ras/MAPK) and reduction of pro‐inflammatory markers (IL‐6, IL‐1β, TNF‐α, MDA, and NO). Consequently, *T. serpyllum* contributes to gut health improvement, neuroprotection, immune modulation, and hepato‐renal and cardio‐pulmonary protection, highlighting its potential medicinal applications.

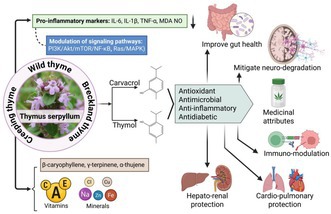

## 1 Introduction

Ethno‐pharmaceuticals and ethno‐nutraceuticals have served humanity since ancient times. Although the modern healthcare system has shifted its reliance on drugs, preventive approaches to promote health have been revitalized in the last few decades. As a result, the application of plants to manage various acute and chronic health problems has gained momentum. Moreover, the demand for herbal medicine has surged due to availability, cost‐effectiveness, and minimal hazards (Khalid et al. 2025). The advanced healthcare system and reformed industrial applications are trying to provide humankind with affordable and accessible healthy approaches. Furthermore, the growing global market of plant‐based medicine and consumer demand for herbal products have manifolded the valuable status of herbs and plants worldwide (Sultan et al. 2023). The thyme genus (Lamiaceae family) is an integral part of traditional herbal medicine practice. *Thymus serpyllum* , a member of the genus thyme, is one such herb highly acknowledged for its medicinal properties. It has been typically used to treat gastrointestinal and respiratory tract issues (cough) and other related health conditions (fever and cold) (Hammoudi Halat et al. 2022). The rich nutritional composition and phytochemistry of *T. serpyllum* enable it to combat obesity, diabetes mellitus, hypertension, cancer, cardiovascular disorders, neurodegenerative diseases, and hepatorenal syndrome. Moreover, *T. serpyllum* displays antioxidant, anti‐inflammatory, and antimicrobial properties, making it an ideal choice for health promotion (Jalil et al. 2024).

Thymol and carvacrol are the most prominent phytochemicals of *T. serpyllum* . Thymol, 2‐isopropyl‐5‐methylphenol (C10H14O), is a monoterpene, colorless, crystalline, distinctly odorized, active constituent of *T. serpyllum* oil. Thymol also occurs in other species of the genus thyme, including *T. vulgaris* , *T. zygis*, and *T. praecox* . Thymol is soluble in alcohol and other organic solvents, but slightly soluble in aqueous medium at neutral pH (Escobar et al. 2020). The studies have been reported that thymol contains antioxidant (Yildiz et al. 2021), anti‐inflammatory (Islam et al. 2024), anti‐microbial (Sharma et al. 2023), antidiabetes (Sachan et al. 2022), anticancer (Taibi et al. 2024), and hepato‐nephroprotection properties (Jamshidi and Taheri 2021; Özmen et al. 2021).

Carvacrol, 2‐Methyl‐5‐(propane‐2‐yl) phenol, an isomer of thymol, also referred as cymophenol, with the chemical formula (C6H3(CH3) (OH)C3H7). It is insoluble in water, but highly soluble in ethanol, CCl_4_, and diethyl ether, with a boiling point of 237.7°C. Carvacrol is abundantly found in *Lavandula multifida*, *Nigella sativa* , *Origanum vulgare* , *Lippia graveolens* , and *Thymus glandulosus* (Imran et al. 2022). The biological properties of carvacrol include antioxidant (Ridaoui et al. 2024), anti‐inflammatory (de Souza et al. 2023), antidiabetes (Hoca et al. 2023), anticancer (Abed et al. 2024), antihypertension (Khazdair et al. 2024), cardioprotective (Joshi et al. 2023), neuroprotective (Forqani et al. 2023), and hepatoprotective and nephroprotective (Cerrah et al. 2023; Najafizadeh et al. 2022).

This review illuminates the therapeutic potential of renowned herbal components with inclusive pharmacological importance. Unlike the earlier reviews, this review is different because it offers insights on *T. serpyllum* , thymol, and carvacrol. This review covers the phytochemistry and nutritional composition of *T. serpyllum* , along with the bioavailability of thymol and carvacrol. The pharmacological properties are the limelight of this review; moreover, this review will shed light on clinical studies, toxicity reports, and industrial applications of thymol and carvacrol. The uniqueness of this review lies in bridging traditional knowledge with modern scientific evidence, emphasizing their role in functional foods, nutraceuticals, and complementary health approaches for sustainable well‐being.

## 2 Methodology

The methodology segment was designed to conduct a review on *T. serpyllum* , thymol, and carvacrol. Various keywords like thymol bioavailability, carvacrol bioavailability, thymol and carvacrol antidiabetes, thymol and carvacrol cardioprotection, thymol and carvacrol and gut health, thymol and carvacrol toxicity, and thymol and carvacrol applications were used to search the data. Boolean operators like (AND, OR) were used to enhance the search. Inclusion criteria: original research articles covering the thymol and carvacrol health‐promoting role, pharmacological aspects, and use. Exclusion criteria: articles other than *T. serpyllum* , thymol, and carvacrol, non‐English articles, and duplicate articles. The most recent studies (2015–2025) were selected for this review; however, some studies are old. The preclinical conclusions were systematically evaluated to analyze the therapeutic potential of thymol and carvacrol.

**FIGURE 1:**
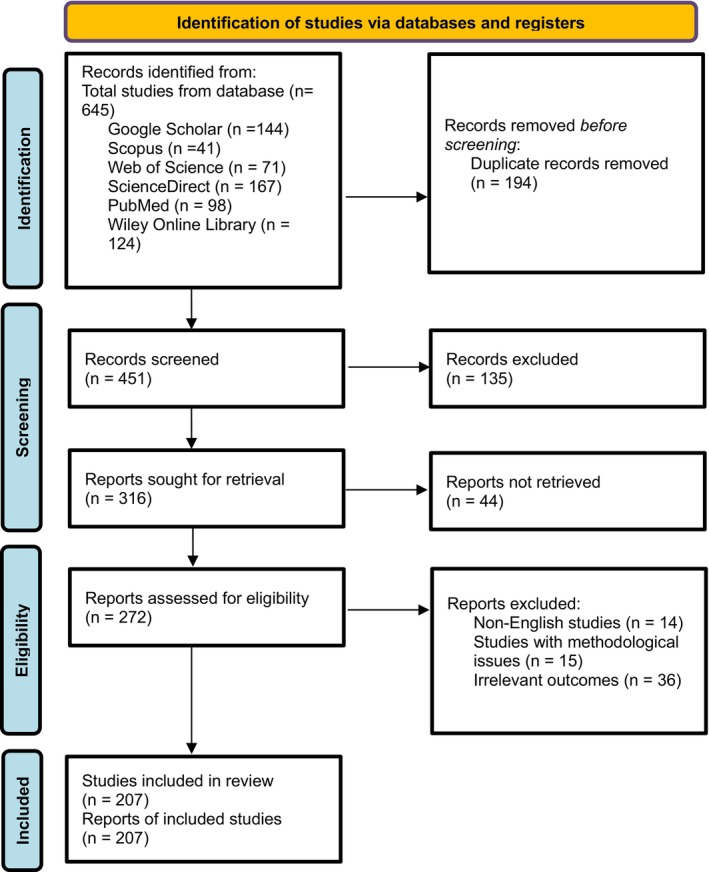
PRISMA 2020 flow diagram of the literature search process.

The study selection for this narrative review followed the PRISMA 2020 flow diagram to ensure a systematic process, as presented in Figure 1. A comprehensive search was conducted on different search engines (Google Scholar, PubMed, ScienceDirect, Scopus, Wiley Online Library, and Web of Science). For Google Scholar, the search was conducted using advanced search with the ‘all in title’ search strategy. A total of 645 studies were initially identified, of which 451 studies remained after removal of duplicates. During initial screening, 135 studies were excluded based on irrelevant titles or abstracts. For the remaining 316 studies, 44 studies could not be retrieved, so only 272 studies were assessed for eligibility. After assessment, 14 studies were excluded due to language, 15 studies were removed as there were methodological issues in those studies, and 36 were excluded for irrelevant outcomes. Hence, only 207 studies were included in the review, including both primary research studies and review articles that met the eligibility criteria. Furthermore, 20 studies referenced solely in the introduction were excluded from the PRISMA diagram as they were not part of the final selection process.

## 3 Habitat, Morphological and Botanical Depiction

**FIGURE 2:**
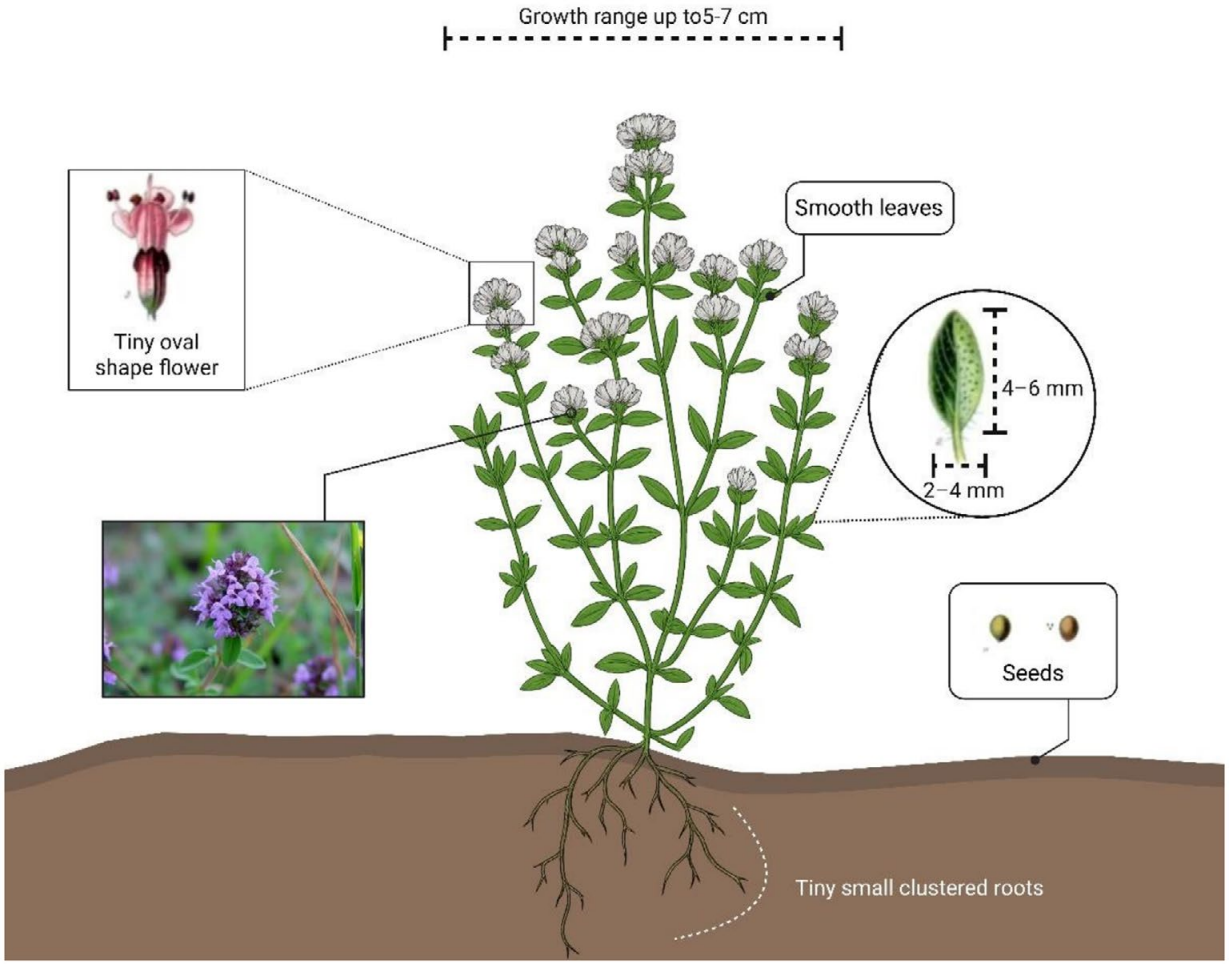
Morphological and botanical depiction of *T. serpyllum* .

*T. serpyllum* , known as wild thyme (WT), creeping thyme, Breckland thyme, Breckland wild thyme, and elfin thyme, is native to several regions of the world, such as Asia, Europe, North America, and East Africa (Jalil et al. 2024). It primarily blooms in high altitudes with loose, rocky, and nutrient‐deprived soil, exhibiting optimal growth under medium to dry moisture conditions and well‐drained circumstances. Moreover, the growth will be maximum in better sunlight. *T. serpyllum* shows notable tolerance to drought and strong winds but tends to reduce in shady zones (Salaria et al. 2023). Figure 2 shows the morphological and botanical depiction of *T. serpyllum* .

It is a perennial shrub that can grow up to 5–7 cm, characterized by its hairy, creeping appearance and clusters of tiny, pink to purple flowers. The oval‐shaped leaves are approximately 4–6 mm long and 2–4 mm wide, have a smooth texture on both sides, are enclosed in long trichomes, and are present throughout the year. The central vein is vigorous, whereas the lateral veins at the base are less visible. *T. serpyllum* exhibits hermaphroditic features and relies on pollinators such as bees, wasps, butterflies, and flies, fascinated by its distinctive fragrance (Jarić et al. 2015). The Taxonomy of *T. serpyllum* is shown in Table 1.

**TABLE 1:** Taxonomy of *Thymus serpyllum* .

Kingdom	Plantae
Phylum	Streptophyta
Class	Equisetopsida
Subclass	Magnoliidae
Order	Lamiales
Family	Lamiaceae
Genus	*Thymus*
Species	*serpyllum*

## 4 Nutrition Profile and Phytochemistry

**FIGURE 3:**
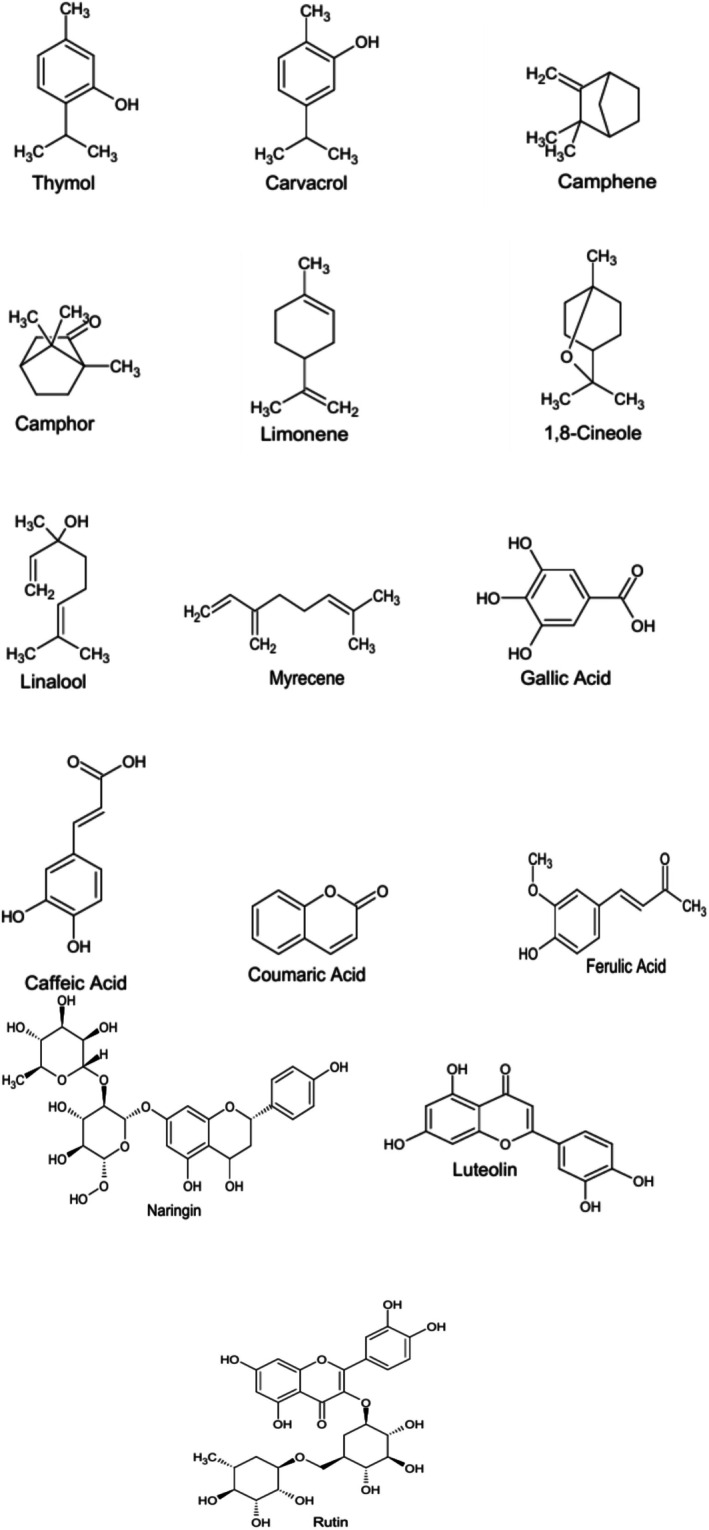
Chemical structures of phytochemicals of *Thymus serpyllum* .

The nutrition profile of *T. serpyllum* revealed that it contains macronutrients (protein, carbs, fat, starch), amino acids, micronutrients like vitamins A, C, E, minerals such as K, Ca, Mg, P, Na, Cl, Fe, S, Zn, Cu, Ni, and dietary fibers (lignin, cellulose, and hemicellulose) (Shahar et al. 2023). Moreover, thyme oil is an exceptional source of folic acid, vitamins A, E, C, and K, as well as other B‐complex vitamins (Dauqan and Abdullah 2017). Phytochemical analysis showed that the reported polyphenols in *T. serpyllum* are gallic acid, caffeic acid, coumaric acid, ferulic acid, catechol, naringin, luteolin, and rutin (Tohidi et al. 2017). The FTIR analysis revealed that it comprises ~11 functional groups, including esters, alcohols, sulfonyl chlorides, alkanes, alkenes, aromatic esters, and halo compounds (Shahar et al. 2023; Cakmakçi et al. 2013). The GC–MS analysis of the methanolic extract revealed a diverse array of phytochemicals, including thymol (1702 mg 100 g^−1^ DW), carvacrol (179 mg 100 g^−1^ DW), linalool (4.97 mg 100 g^−1^ DW), and limonene (4.97 mg 100 g^−1^ DW) (Sonmezdag et al. 2016). In addition, essential oil consists of carvacrol (37.49%), β‐caryophyllene (6.51%), camphene, limonene (1.8%), 1,8‐cineole, γ‐terpinene (10.79%), linalool, camphor, α‐thujene, and myrcene (3.8%) (Aćimović et al. 2019). The phytochemicals of *T. serpyllum* are shown in Figure 3, while nutritional and phytochemical composition are depicted in Tables 2 and 3, respectively.

**TABLE 2:** Nutritional composition of *T. serpyllum* .

Composition	Nutrient	Quantity
Proximate Analysis (g/100 g)	Protein	21.39
	Carbohydrate	11.85
	Fat	5.54
	Ash	2.73
	Starch	6.52
	Amino Acid	1.99
Vitamins (mg/100 g)	A	4.90
	C	43.8
	E	13.7
Minerals (mg/100 g)	K	2100
	Ca	1850
	Mg	430
	P	200
	S	170
	Al	280
	Na	20
	Cl	120
	Si	610
	Fe	160
	Zn	3.8
	Mn	9.6
	Cu	9.6
	Ni	0.8
Dietary fibers (g/100 g)	NDF	15.05
	ADF	10.64
	Lignin	3.58
	Hemicellulose	4.15
	Cellulose	7.06

*Source:* Shahar et al. (2023).

**TABLE 3:** Phytochemical composition of *T. serpyllum* .

Extract	Volatile compounds	Concentration (mg 100 g^−1^ DW)
Methanolic	α‐Pinene	18.1
	Camphene	7.68
	3‐Penten‐2‐ol	7.81
	Myrcene	28.4
	Limonene	5.99
	γ‐Terpinene	90.4
	p‐Cymene	88.8
	Borneol	7.32
	Linalool	4.97
	Carvone	5.54
	Thymol	1702
	Carvacrol	179
	Hexadecanoic acid	5.99
	**Phenolic compounds**	**Quantification (mg g** ^**−1**^ **dry herb)**
	Gallic acid	0.63
	Caffeic acid	1.73
	Chlorogenic acid	7.88
	Naringin	3.03
	Ferulic acid	4.54
	Rutin	1.35
	Rosmarinic acid	21.72
	Luteolin	48.04
	Kaempferol O‐glucuronide	15.21
	Apigenin O‐glucuronide	1.93

*Source:* Sonmezdag et al. (2016).

## 5 Bioavailability of Thymol and Carvacrol

The bioavailability of any compound is a crucial factor in achieving its maximum health benefits, and this process involves various pathways/steps. The food source is one of the key factors because the content/concentration of the bioactive compounds in that particular source is vital. Along with source and concentration, the form/texture of the compound, delivery route, vehicle, and GIT conditions are other prime factors affecting the compound's digestion, absorption, and bioavailability (Noman, Sultan, Maaz, Mazhar, et al. 2025). The intestine is an important location for thymol absorption and biotransformation. The intestinal absorption occurs through passive diffusion, the intestinal lymphatic system, and rapid upper gut absorption. Thymol, being a tiny, lipophilic, acidic molecule, easily passes the intestinal epithelial membranes. However, when incorporated into lipid‐based formulations, it mixes with chylomicrons and moves into lymphatic vessels. Lastly, the unprotected thymol is speedily absorbed in the duodenum, resulting in inadequate concentrations for the distal gut (Kristofova et al. 2025). Thymol and its metabolites are processed in the intestinal wall, transformed into hydrophilic metabolites, like thymol sulfate and thymol glucuronide; thus, they undergo two possible fates after biotransformation: they can either be transported back into the intestinal lumen or changed back into parent compounds and redistributed within the organism. However, a few parts of the compounds are transported by the mesenteric vein into the liver, metabolized, and then evacuated into the duodenum in bile, where they are reabsorbed (Bacova et al. 2020). The constituents pass through some barriers during their metabolic pathway in organisms, hindering their absorption. The first step is metabolism in the intestine, which reduces the number of constituents reaching the bloodstream, and then metabolism in the liver constitutes the second barrier for distribution throughout the organism. Furthermore, numerous efflux transporters are attached to lipophilic components that are quickly eliminated from the organism, substantially restraining their bioavailability (Placha et al. 2022). In that regard, van Noten et al. (2022) studied the in vitro stability and ex vivo absorption of thymol α‐D‐glucopyranoside (TαG) and thymol β‐D‐glucopyranoside (TβG) in the gastrointestinal system of piglets. They determined that there were no changes in TαG and TβG in the mucosa between the beginning and end of the absorption measurement. Nevertheless, TβG was more degraded in the intestine through hydrolysis by bacteria, and TαG was less susceptible to degradation. Bacova et al. (2021) investigated the level of biotransformation and concentration of thymol (250 mg/kg feed) in rabbits. They determined that the thymol level was greater in the intestinal walls compared to the liver and plasma. Moreover, histopathological examination revealed that there was a higher concentration in the kidney than in the liver, indicating that the kidney experiences maximum metabolism. In another study, the topical application of thymol and carvacrol to cure mastitis in dairy animals revealed that both compounds were present in plasma, milk, liver, and fat (Mason et al. 2017). Taken together, the oral administration of thymol undergoes several metabolic phases, particularly hepatic biotransformation via phase I oxidation and phase II conjugation, later distributed to different vital organs through the bloodstream and then eliminated by the body. On the other hand, topical applications result in higher bioavailability due to least systemic exposure, therefore substantially affecting therapeutic potential.

A study on broiler chickens identified thymol and metabolite concentrations in the plasma, liver, and duodenal wall of chickens (Pisarčíková et al. 2017). Likewise, Ocel'ova et al. (2016) reported thymol content in the blood plasma, liver, and muscle of chickens. Haselmeyer et al. (2015) assessed the concentration of thymol (0%, 0.1%, 0.2%, 0.3%, and 1%) in the gut, blood plasma, liver, and muscles. They concluded that a 1% thymol concentration was maximum in the gut and plasma rather than in the organs and muscles. The poor bioavailability of thymol or carvacrol results in rapid loss, but also hinders their clinical significance. However, novel strategies such as nanoencapsulation can improve their stability, bioavailability, and bioactivities. In this context, Zamani et al. (2015) encapsulated thymol with hydroxypropyl methyl cellulose to enhance the duration of thymol action. Moreover, it has been found that thymol and methylcellulose encapsulation remarkably improve the bioavailability of thymol compared to free thymol (Rassu et al. 2014). In conclusion, nanodelivery of thymol and carvacrol by using different nano‐materials can enhance their stability due to their improved water solubility and resistance towards degradation.

## 6 Antioxidant Capacity

An imbalance of reactive oxygen species can cause oxidative stress, DNA damage, and genetic mutations. Bioactive compounds can neutralize these species, attenuate inflammation, and reduce the risk of chronic diseases (Arif et al. 2024). *T. serpyllum* , thymol, and carvacrol have been reported to have significant antioxidant activity. Mrkonjić et al. (2024) investigated novel extraction techniques along with WT's in vitro antioxidant potential using DPPH and ABTS+ assays, and concluded that ABTS+ exhibited a higher antioxidant capacity than DPPH. Degenek et al. (2023) determined the antioxidant activity of fresh kombucha cheese fortified with *T. serpyllum* . They demonstrated that total phenols content improved in all samples, particularly those fortified with dry *T. serpyllum* extract with the value of 1.86 ± 0.01 to 2.14 ± 0.01 mg GAE/g, and the antioxidant capacity of the sample with ground WT was amplified in the FRAP assay and the recorded values are 13.8 ± 0.00 to 15.10 ± 0.00 μM Fe^2+^/g. Aksić et al. (2023) assessed TFC, polyphenols, and antioxidant activity in various extracts (ethanolic, chloroform, and ethyl acetate). They concluded that the ethanolic extract proved to be the best, with the highest polyphenol and TFC yields, and the highest antioxidant activity in the DPPH assay. Pavlić et al. (2022) used natural deep eutectic solvents to enhance antioxidant capacity and polyphenolic yield in DPPH, ABTS, and FRAP assays. Different temperatures (40°C, 55°C, and 70°C), varying extraction times (60, 120, and 180 min), and L/S ratios (10, 20, and 30 g sample) were employed. It was found that 65°C, 180 min, and an L/S ratio of 28 g were the optimal conditions for extracting the maximum yield.

New technologies, including nanoencapsulation/nanoparticles (NPs), have been shown to be excellent approaches to achieving the desired results. Thymol‐chitosan‐gelatin (TCG) NPs showed better antioxidant capacity and stability and had a greater antioxidant activity in FRAP, ABTS, and DPPH assays at pH (5.5 and 7.0) and 4°C and 25°C (Ojeda‐Piedra et al. 2024). Yildiz et al. (2021) reported that the antioxidant activity of carvacrol, thymol, and thymoquinone, which enhance the stability of refined and stripped corn oils, was assessed by CD (K232), Rancimat, peroxide value, and p‐anisidine value methods. Ramos et al. (2020) reported that thymol‐based polylactic acid (PLA) loaded silver nanocomposite films exhibited antioxidant activity in the DPPH assay and antibacterial activity. Furthermore, Ruiz‐Malagón et al. (2022) reported the antioxidant potential of *T. serpyllum* in obese mice through attenuating inflammation and regulating gut dysbiosis. Additionally, studies on carvacrol have proved its antioxidant capacity in both in vivo and in vitro models, accredit to the presence of the –OH, linked to the aromatic ring (Aristatile et al. 2015). Carvacrol not only showed antioxidant activity but also ameliorated metabolic disorders‐linked adverse effects. In this context, carvacrol (15 mg/kg body) reduced MDA and improved CAT, SOD, and GPx in diabetic rats (Tabibzadeh Dezfuli et al. 2017). Conclusively, both thymol and carvacrol have shown in vitro and in vivo antioxidant activity; however, antioxidant potential can vary depending on assay type, solvents, and in vivo experimental conditions.

## 7 Health Promoting Perspectives

*T. serpyllum* , thymol, and carvacrol have been used for several health benefits and the treatment of numerous ailments worldwide. Different parts of *T. serpyllum* , especially leaves, have been widely used in traditional medicine systems. Table 4 depicts the health‐promoting attributes of *T. serpyllum* , thymol, and carvacrol via possible pathways/mechanisms.

**TABLE 4:** Health promoting attributes of *T. serpyllum* , thymol and carvacrol.

	Form/dosage	Study type	Animal/cell line	Property	Results/technique	References
*T. serpyllum*	EO	In vitro	*Candida albicans*	Antifungal	Disk‐diffusion, serial dilution, MIC (0.49–3.9 μg/mL)	Shapoval et al. (2023)
	EO		SCC‐25	Cytotoxicity	**↓**Proliferation in SCC‐25 cell line	Lazarević et al. (2019)
	Extract		MCF‐7 and MDA‐MB‐231	Anticancer	**↓**DNMT and HDAC activities, **↑**caspase 3/7 activity	Bozkurt et al. (2012)
	EO		*Pseudomonas aeruginosa*	Antibacterial	Modification of protein profiles of bacteria, MIC (0.134 ± 0.09 mg/mL)	Kačániová et al. (2024)
	Methanolic extract	In vitro and in vivo	BALB/C mice	Anti‐inflammatory	**↓**Src tyrosine kinase activity, IL‐6	Kindl et al. (2019)
	Aqueous extract (500 and 800 mg/kg/d)	In vivo	BALB/C mice	Antidiabetic	**↑**IRS1 and GLUT2 gene and AMPK expression	Azhar et al. (2022)
	100 mg/kg BW		Wistar rats	Antihypertension	**↓**Systolic and diastolic BP, total peripheral resistance in SHR	Mihailovic‐Stanojevic et al. (2013)
	Aqueous extract (500 mg/kg)		Rabbits	Hepatoprotection	**↓**ALT, AST, ALP, **↑**GSH, GPx	Mushtaq (2017)
	Extract		Healthy but overweight humans	Gut health	**↑**Stool frequency, gut health	Knaub et al. (2022)
Thymol	11 μM	In vitro	HepG2	Anticancer	**↓**TOS, **↑**TAS	Altintas et al. (2023)
	1.25 mg/mL		*E. sakazakii*	Antibacterial	Morphological changes in *E. sakazakii* , MIC (1.25 mg/mL)	Tian et al. (2021)
	Thymol‐pyrazole hybrid		COX‐2 and 5‐LOX enzymes	Anti‐inflammatory	**↓**COX‐2/5‐LOX	El‐Miligy et al. (2023)
	40 mg/kg BW	In vivo	Wistar rats	Antidiabetic	**↓**TC, TG, LDL, blood glucose	Agarwal et al. (2020)
	80 mg/kg BW		BALB/c mice	Hepatoprotective	**↓**ALT, AST, ALP, TNF‐α, IL‐6	Dou et al. (2022)
	10, 30, 50 mg/kg		Male rats	Nephroprotective	**↑**SOD, GPx	Jamshidi and Taheri (2021)
	20 and 40 mg/kg		C57BL/6 J mice	Neuroprotective	**↓**PI3K/Akt/mTOR/NF‐κB	Zhao et al. (2024)
	100, 200, and 400 mg/kg		Guinea pigs	Asthma	**↓**Number of cough bouts, antitussive and muco‐suppressant effects	Ozolua et al. (2016)
	100 mg/kg		Wistar rats	Gastric ulcer	**↑**Mucus secretion, ATP‐sensitive K^+^ channels	Ribeiro et al. (2016)
	0.4% thyme		Balb/c mice	Gastrointestinal	**↓**IL‐1*β*, IL‐6, TNF*α*	Bukovská et al. (2007)
Carvacrol	Carvacrol, citral and 2‐(E)‐hexenal	In vitro	*E. coli*	Antibacterial	DNA microarray technology, downregulation of genes	Siroli et al. (2018)
	0–200 μg/mL		4 T1 and L929	Anticancer	**↑**Caspase3, apoptosis	Jamali et al. (2020)
	10 to 50 μM		3 T3‐L1 and WJ‐MSCs	Anti‐obesity	**↓**Adipogenic differentiation, ChREBP expression in 3 T3‐L1 cells	Spalletta et al. (2018)
	50 mg/kg	In vivo	Sprague–Dawley rats	Neuroprotection	**↓**TRPM7 channels, oxidative damage, degenerating neurons	Hong et al. (2018)
	50, 100 and 200 mg/kg		Sprague–Dawley rats	Hepatoprotective	**↑**SOD, GPx, CAT, **↑**AST, ALP, MDA	Bakır et al. (2016)
	10, 20 mg/kg		C57BL/6 J mice	Antidiabetic	**↑**Ki67 expression, **↓**PI3K/Akt	Liu et al. (2020)
	10 and 50 mg/kg		Mice	Anti‐inflammatory	**↓**IL‐2, IFN‐γ, IL‐10, IL‐17	Rolim et al. (2019)

## 8 Anticancer Activity

**FIGURE 4:**
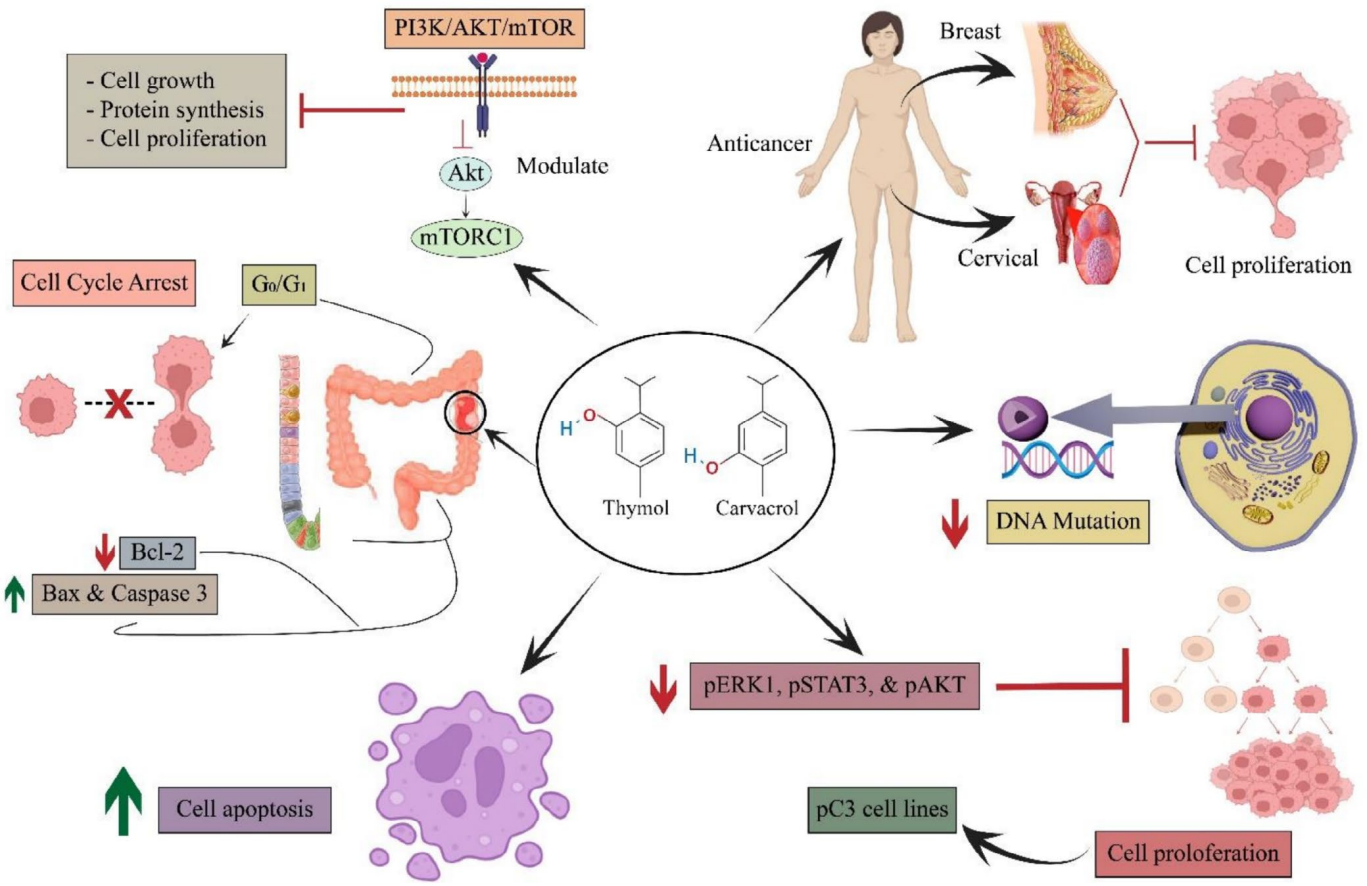
Anticancer activity of thymol and carvacrol via cell proliferation inhibition, apoptosis induction, Bcl‐2 downregulation, vimentin, α‐SMA MMP‐2/9 inhibition, caspase 3 upregulation, Wnt/β‐Catenin, PI3K/AKT/ERK, and PI3k/Akt/mTOR pathway modulation, pERK1/pSTAT3 suppression, and cell cycle arrest at G_0_/G_1 p_hase.

Cancer, a leading global cause of death, arises from complex mechanisms involving multiple pathways. Inflammation and OS play key roles, triggered by infections, toxins, chemicals, heavy metals, radiation, food components, and over the counter (OTC) drugs. These factors disrupt the ROS/RNS balance, induce genetic mutations, initiate uncontrolled cell proliferation and metastasis, and ultimately drive oncogenesis (Noman, Sultan, Mazhar, Baig, et al. 2025). Several in vitro and in vivo studies of *T. serpyllum* , thymol, and carvacrol have proven anti‐inflammatory and anticancer potential against various cancers. The association of PI3K/AKT/mTOR with cancer progression has been often discussed in various studies. It is a crucial signaling pathway involved in cell growth, proliferation, and several metabolic mechanisms. This pathway activates due to the binding of an external signal like EGF with receptor tyrosine kinase, which then activates PI3K and PIP3. The activation of PIP3 recruits AKT and phosphorylated via PDK1, thus triggering mTOR, a key regulator of cell development (Cerma et al. 2023). Thymol can inhibit cell growth through apoptosis and cell cycle arrest, modulate the PI3K/mTOR/ERK/AKT signaling pathways, and enhance the effectiveness of 5‐FU (Herrera‐Bravo et al. 2024). Alam et al. (2021) reported thymol‐based anticancer activity of 1, 2, 3‐triazole hybrids in MCF‐7 and MDA‐MB‐231 breast cancer cell lines. They concluded that thymol‐derived compounds, especially compound 10 (10 μM), exhibited 3.2 times better anticancer activity than 5‐FU in MCF‐7 cells and 1.42 times better growth inhibition than tamoxifen in MDA‐MB‐231 cell lines. In another study, *Thymus capitatus* essential oil (EO) (0.5%, 1%, and 2%), comprising thymol as a major component, demonstrated apoptotic effects in colon stem cells by activating caspase‐3. They found that a 0.5% dilution of *T. capitatus* EO proved more effective in reducing cell proliferation than 1% and 2% dilutions (Yavuz et al. 2021). The Wnt/β‐Catenin pathway is often associated with colorectal cancer (CRC) development, and studies have shown that it could be a potential target for ameliorating CRC. Regarding this, Zeng et al. (2020) demonstrated that thymol (75 and 150 mg/kg) exhibited an oncoprotective effect against HCT116 and LoVo CRC cell lines via reducing tumor growth, upregulating caspase‐3, modulating the Wnt/β‐Catenin pathway, and downregulating Bcl‐2 in BALB/c nude. Moreover, in an in vitro assay, thymol (0, 10, 20, 40, 80, or 120 μg/mL) reduced cell metastasis and EMT in HCT116 and LoVo cells with an IC_50_ of 46.74 μg/mL and 41.46 μg/mL, respectively. In conclusion, thymol showed better anticancer activity against LoVo cell lines with a low IC_50_ value. Thymol‐based studies have shown that thymol can inhibit K‐562, HL‐60, and gastric cancer cell lines via cell cycle arrest at the G0/G1 stage (Kang et al. 2016; Jaafari et al. 2012; Deb et al. 2011). Furthermore, thymol (0.5, 1, 2, 4 mM) inhibited cell migration in HT‐29 colon cancer cell lines through the downregulation of the PI3K/AKT/ERK pathway. Additionally, the results demonstrated that thymol reduced MMP‐2/9 activity, improved E‐cadherin expression, and inhibited vimentin and α‐SMA in HT‐29 cells (Lv and Chen 2017). Bansal et al. (2022) investigated the in vitro cytotoxicity of carvacrol derivatives on the A549 and BALB‐3 T3 lung cancer cells. The results showed that a 500 μg/mL concentration results in cell migration inhibition and cell cycle arrest at G2/M phase. It has been proven that 2‐cyclodextrin, a water‐soluble cyclic oligosaccharide, is used in drug delivery to improve drug stability and bioavailability. Regarding this, carvacrol (25–200 μg/mL) and 2‐cyclodextrin (200 μg/mL) complex inhibited PC3 cell line migration and invasion, and it was found that the carvacrol/2‐cyclodextrin complex (100 μg/mL) showed the highest anticancer potential (Trindade et al. 2019). In another study, carvacrol (0–800 μM) suppressed pERK1/pSTAT3 and pAKT expression and exhibited an antiproliferative effect on PC3 cell lines. The study concluded that the IC_50_ of carvacrol was 360 μM for PC3 cell lines (Heidarian and Keloushadi 2020). Besides the conjugation approach, nanodelivery could be a potential technique to enhance drug efficiency in oncotherapy. Carvacrol‐chitosan NPs with doxorubicin showed anticancer synergistic effects in cisplatin‐resistant cervical (HeLa) and breast (MCF‐7) cancer cells. It was discovered that the combination was effective in lowering cell proliferation and growth with an IC_50_ of 8.47 μg/mL and 5.66 μg/mL against MCF‐7 and HeLa cell lines, respectively (Akhlaq et al. 2023). In a nutshell, both thymol and carvacrol have antiproliferative and anticancer potential by inducing apoptosis, cell cycle arrest, and modulating molecular pathways. However, their low bioavailability can hinder their pharmacological potential; thus, novel methods like nanodelivery and conjugation with other components are suitable approaches to improve stability, bioavailability, and control drug release. The anticancer activity of thymol and carvacrol is presented in Figure 4.

## 9 Hypoglycemic and Antidiabetic Potential

Diabetes mellitus is a fatal metabolic disorder affecting nearly 25% of the global population, characterized by hyperglycemia due to insufficient insulin synthesis or insulin resistance. Main risk factors include obesity, smoking, lifestyle habits, genetics, and toxins. In addition, diabetes leads to complications such as hypertension, stroke, cancers, neuropathy, nephropathy, and diabetic foot, damaging vital organs (Noman, Sultan, Mazhar, Khan, et al. 2025).

The antidiabetic and hypoglycemic effects of medicinal herbs are credited to their rich phytochemistry and have been proven in earlier studies (Arif et al. 2024). Similarly, Shittu et al. (2024) investigated the antidiabetic activity of thymol (20 mg/kg) in Wistar rats. The study proved that thymol reduced glucose levels, TGs, and LDL by reducing pancreatic α‐amylase activity. In another study, thymol oral administration (25 mg/kg) and topical (0.5% thymol gel) application reduced oxidative stress and diabetes markers (IL‐1α, TC, TGs, MDA, H_2_O_2_, and blood glucose) in volunteers (Martirosyan et al. 2022). Sachan et al. (2022) evaluated the antidiabetic activity of thymol (10 and 20 mg/kg) in STZ‐induced diabetic neuropathy rats and concluded that thymol reduced TNF‐α, NO, and LPO, while improving SOD expression. Thymol supplementation (40 mg/kg) showed antidiabetic property in STZ‐induced diabetic rats. The study findings revealed that the blood glucose, LDL, urea, creatinine, TGs, and TC, ALT, AST, and ALP significantly reduced, and thymol supplementation improved SOD and GSH levels (Agarwal et al. 2020). In another study, Jia et al. (2018) showed the hypoglycemic effect and effectiveness of carvacrol in relation to insulin‐resistance‐induced heart disease. Moreover, Hou et al. (2019) confirmed the effectiveness of carvacrol in treating diabetic cardiomyopathy through the regulation of the PI3K/AKT/GLUT4 pathway in mice. Additionally, carvacrol (20 mg/kg BW) reduced hyperlipidemia and hyperglycemia in the C57BL/6J mice by enhancing the insulin sensitivity and glucose metabolic enzymes (Ezhumalai et al. 2014). Table 5 presents the hypoglycemic and antidiabetic role of thymol and carvacrol.

**TABLE 5:** Hypoglycemic and antidiabetic potential of thymol and carvacrol.

	In vivo (animals)	Dosage/duration	Mechanism	References
Thymol	C57BL/6J mice	40 mg/kg	**↓**TGF‐β1, VEGF, lipid peroxidation	Saravanan and Pari (2016)
	C57BL/6J mice	10, 20, 40 mg/kg	**↓**TG, LDL, TC, LDL, **↑**HDL, lipid enzyme activity	Saravanan and Pari (2015)
	Mice	40 mg/kg	**↓**TGF‐β1, SREBP‐1c expression	Aman et al. (2013)
	Wistar rats	14 mg/kg twice/day	**↓**ALT, AST, glucose, BUN, leptin levels	Haque et al. (2014)
Carvacrol	Wistar rats	75 mg/kg	**↓**MDA, Bax, germ cell apoptosis	Shoorei et al. (2019)
	Sprague–Dawley rats	50 mg/kg	**↑**GPR41/43 expression, **↓**glucose levels	Sun et al. (2024)
	Wistar rats	25, 50, and 100 mg/kg	**↓**TNF‐α, NF‐κBIL‐1β, MDA, **↑**SOD, GSH, caspase‐3	Deng et al. (2013)
	Wistar rats	75 mg/kg	**↓**MDA, Bax, COX‐2, **↑**SOD, GSH, CAT	Arkali et al. (2021)

## 10 Immunomodulatory Impact

The immune system protects against pathogens and inflammation through a network of cells, proteins, and signaling mediators. It comprises three types: innate (first defense) and acquired (second defense), together warranting effective protection and immune regulation (Munteanu and Schwartz 2022). The immunomodulatory effects of thymol and carvacrol have been reported in various studies. Li et al. (2023) demonstrated that thymol inhibits IL4I1 expression, blocks aryl hydrocarbon receptor signaling, and enhances the efficacy of anti‐PD‐1 antibodies in lung adenocarcinoma cells. Thus, thymol can prove effective in immunotherapy against LUAD. Thymol (1 or 2 g/kg diet) inhibited ZnO‐NPs immunotoxic effects in fish via modulating serum immunoglobulins (Igs) (Khalil et al. 2023).

Encapsulated thymol and carvacrol combination (150 mg/kg) improved SOD, GSH‐Px, and Nrf2 mRNA levels while suppressing NF‐κB and IL‐1β in broilers (Li et al. 2022). Stojanović et al. (2021) concluded that thymol (50 and 100 mg/kg) proved effective in inducing an immune‐modulatory impact against L‐arginine‐induced acute pancreatitis PE cells via decreased PEC mitochondrial activity, MPO and ROS levels, and α‐amylase activity. Favaretto et al. (2020) micro‐encapsulated carvacrol, thymol, and cinnamaldehyde, supplemented (500, 1000 mg phytogenic/kg) to lambs and reported improved antioxidant and anti‐inflammatory enzymes.

Carvacrol diminished colonic epithelial apoptosis, inhibited pro‐inflammatory immune response, mitigated acute campylobacteriosis, and reduced the risk for infectious complications in human gut microbiota‐linked IL‐10−/− mice (Heimesaat et al. 2024). Liu, Kong, et al. (2022) demonstrated that carvacrol concentrations (150, 300, and 450 g/t) significantly improved GSH‐Px, reduced diarrhea, enhanced digestive enzymes, and increased the thymus index in rabbits. Zheng et al. (2021) fed 50 mg/kg carvacrol to influenza virus A‐infected C57BL/6 mice and concluded that carvacrol mitigated lung tissue injury, inhibited IFN‐γ, TNF‐α, IL‐12, IL‐1, and IL‐6 expression, suppressed IRF mRNA, MyD88, NF‐κB, IPS‐I, IRAK4, RIG‐I, TLR7, and TRAF6 levels. A study on LPS‐challenged broilers demonstrated that carvacrol regulated TNF‐α, NF‐κB p65, IL‐6, TLR4, TLR2, and SIgA, thereby proving effective in immunomodulation via TLRs/NF‐κB and displaying an anti‐inflammatory function (Liu et al. 2019).

## 11 Hepato‐Renal Protective Effects

The liver and kidneys are vital organs that are associated with numerous biochemical mechanisms, including metabolism, detoxification, reabsorption, secretion, and the synthesis of proteins, enzymes, and hormones. Injury to these key organs leads to impaired function, disturbed biochemical reactions, organ failure, and even death. OTC drugs, unhealthy food intake, and environmental chemicals and toxins are major factors contributing to hepato‐renal toxicity and damage (Motawi et al. 2020). Thymol and carvacrol, as terpenoids, undergo three primary UPR pathways in response to ER stress, and these compounds protect the liver from assaults by modulating PERK, IRE1, and ATF6 signaling pathways (Zhang et al. 2022). Recently, Peirovy and Asle‐Rousta (2024) studied the impact of thymol (10 mg/kg) and p‐cymene (50 mg/kg) on liver injury in rats. They reported that both compounds reduced IL‐6, TNF‐α, NF‐κB, and improved Nrf2 and HO‐1 expression. Maleki et al. (2024) administered thymol (30, 60, and 90 mg/kg) to mice experiencing morphine withdrawal syndrome and found that thymol improved hepatic function by lowering ALT, ALP, and AST while enhancing SOD, GSH, and CAT activity. Zinc oxide NPs have been proven toxic to fish and have reduced their performance. However, thymol (1 or 2 g/kg diet) enhanced SOD, GSH, CAT, HSP‐70, caspase‐3, and P53 expression and decreased MDA levels (Abou‐Zeid et al. 2023). A study by Lahmi et al. (2023) explored the hepatoprotective role of thymol (50 mg/kg) and vitamin E (200 mg/kg) in NAFLD rats, finding that both treatments reduced levels of TNF‐α, p53MAPK, CK‐MB, ALT, and AST. Esmaeili et al. (2024) investigated the hepatoprotective effect of carvacrol (25, 50, or 100 mg/kg) against thioacetamide‐induced liver injury and encephalopathy in rats and reported decreased lipid peroxidation and improved activity of antioxidant enzymes. Cerrah et al. (2023) reported that carvacrol (50 mg/kg) alleviated acrylamide‐hepatotoxicity by reducing MDA, TNF‐α, ALT, AST, IL‐1β, and NF‐κB, and improving TAS.

**FIGURE 5:**
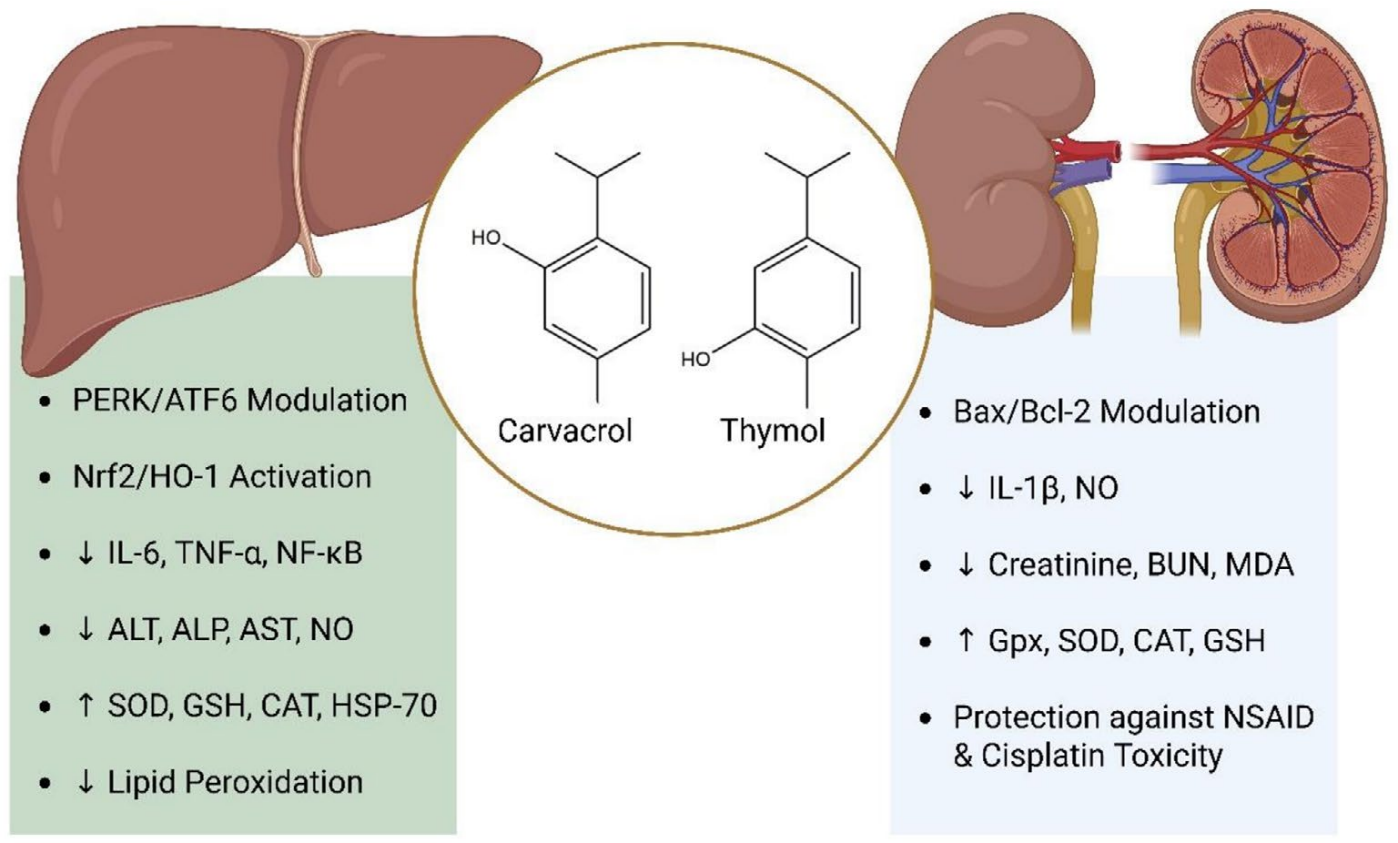
Hepato‐renal protective role of thymol and carvacrol via upregulation of SOD, GSH, CAT, and HSP‐70, downregulation of IL‐6, TNF‐α, NO, and lipid peroxidation, NF‐κB, Nrf2/HO‐1 activation, reducing BUN, ALT, ALP, AST, MDA, and creatinine, and modulation of PERK/ATF6 and Bax/Bcl‐2.

Renal injury or damage results in impaired kidney function and may lead to renal failure. However, carvacrol has proved effective in recovering from renal injuries. Mortazavi et al. (2024) examined the effect of carvacrol on IL‐1β and NO in renal injury. They stated that carvacrol (25, 50, 100 mg/kg) reduced serum creatinine, BUN, NO, and IL‐1β. Najafizadeh et al. (2022) proved that carvacrol (10 mg/kg) reduced MDA, BUN, enhanced SOD, GPx, and modulated Bax, Bcl‐2, and caspase‐3 expression. It has been reported that non‐steroidal anti‐inflammatory drugs are associated with liver injury, and carvacrol has been found to alleviate creatinine, BUN, MDA, and TNF‐α, while enhancing GPx, CAT, SOD, and GSH activity (Nouri et al. 2021). Salari et al. (2021) formulated carvacrol‐loaded β‐cyclodextrin‐alginate‐chitosan NPs to examine their therapeutic effect against malathion and parathion‐induced renal injury and stated decreased renal biomarkers. Jamshidi and Taheri (2021) reported the nephroprotective role of thymol, which also improved SOD, GPx, and GSH. Thymol (10, 20 mg/kg) can attenuate oxidative damage and lower elevated renal biomarkers (Baldissera et al. 2018). Moreover, thymol (20 mg/kg) and carvacrol (15 mg/kg) inhibited cisplatin‐induced renal injury by alleviating OS and inflammation in Swiss Albino rats (Em et al. 2015). Figure 5 presents the hepatorenal protective effect of thymol and carvacrol.

## 12 Thymol, Carvacrol and Gut Health

The gastrointestinal tract (GIT) plays a pivotal role in digestion, absorption, metabolism, and nutrient distribution. However, gut health faces challenges from infections, poor dietary habits, toxins, and environmental chemicals. Disturbed gut microbiota causes dysbiosis, leading to inflammation, impaired function, and chronic health problems (Parkin et al. 2021). The interlink between NF‐κB and gut dysbiosis involved a complex chain of inflammatory markers activation. Being a master regulator, NF‐κB activates various cytokines like IL‐6, IL‐1β, and TNF‐α to trigger inflammation in gut dysbiosis. However, microbial metabolites such as UroA promote the Nrf2 pathway to enhance gut barrier function and reduce inflammation (Singh et al. 2019). Moreover, immune cells (macrophages, T and B cells), particularly T cells, have been extensively influenced by gut microbiota and SCFAs. Butyrate, a SCFA, regulates T cells function via hindering histone deacetylases, thus leading to Treg differentiation and IgA production to attenuate inflammation (Luu et al. 2021). Thymol and carvacrol both have proven effective in maintaining gut integrity. It has been found that carvacrol, when combined with treatment regimens, attenuates acute campylobacteriosis, reduces IFN‐γ, inhibits neutrophils and T lymphocytes, and mitigates diarrheal symptoms in IL‐10−/− mice (Foote et al. 2023). Xu et al. (2023) reported that a thymol and carvacrol mixture (200 mg/d) modulated gut microbiota, increased *Bifidobacterium* spp. and *Akkermansia* spp., reduced *Enterobacteriaceae*, and amplified intestinal butyric acid, thereby diminishing diarrhea and pro‐inflammatory markers in lambs. Toll‐Like Receptors, specifically TLR4, are overactivated in gut dysbiosis due to excessive LPS production, which binds with TLR4 to produce pro‐inflammatory cytokines and trigger inflammation (Chen et al. 2024). However, carvacrol has the potential to modulate TLR expression to alleviate dysbiosis and in this context, Wu, Ma, et al. (2023) proved that carvacrol (25 g/t) alleviated intestinal inflammation, downregulated TLR gene expression, and mitigated gut dysbiosis in LPS‐challenged rabbits. In another study, carvacrol, combined with menthol and carvone (150 g/t), effectively reduced intestinal pathogens, improved gut microbiota, and alleviated inflammation in commercial layers (Bajagai et al. 2022). Wang et al. (2022) reported that thymol and carvacrol (100 mg/kg) improved gut microbiota, enhanced egg quality, and increased intestinal integrity.

Thymol supplementation (0, 100, 200, 300 mg/kg) significantly improved organic matter and crude protein digestibility, and improved IgA, IgM, VH of the ileum and duodenum, CD of the jejunum. Moreover, 100 mg/kg thymol upsurged IL‐4 and CAT, and overall, thymol improved the population of *Bifidobacterium*, *Fusobacterium*, and *Lactobacillus* in the gut of blue foxes (Yuan et al. 2024). Thymol, administered at various doses, has proven efficient enough to improve gut conditions and alleviate bacterial infections in multiple animal studies (Anderson et al. 2021; Toschi et al. 2020). Studies have shown that the combination of phytochemicals proved more effective in amelioration of disease rather than a single compound. In this concern, thymol (50, 100, 200, and 500 mg/kg) along with oleuropein (50–200 mg/kg) proved effective in treating indomethacin‐induced gastric ulcers. However, 500 mg/kg was aversive to the conditions, while the rest of the treatments reduced eNOS, TNF‐α, and PGE_2_, modulated the TAC/TOS balance, and attenuated caspase‐3, thereby reducing inflammation in Sprague–Dawley rats (Koc et al. 2020). Ribeiro et al. (2016) reported the gastroprotective role of thymol (100 mg/kg) in both acute and chronic gastric ulcers, mediated by enhanced mucus secretion and activation of ATP‐sensitive K+ channels.

## 13 Cardio‐Pulmonary Protective Role

The cardiovascular and pulmonary systems primarily consist of the heart and lungs, two major organs that play a crucial role in blood and air circulation throughout the body. Any disturbance in these two results in irregular function and may even cause death. The roles of thymol and carvacrol in addressing cardio‐respiratory problems have been documented. Carvacrol (25, 50, and 75 mg/kg) reduced BP, heart rate, and MDA in vivo, whilst hypertrophy decreased in H9c2 cardiomyoblasts in vitro. Thus, carvacrol proved effective against cardiac hypertrophy in both in vivo and in vitro models (Jamhiri et al. 2019). The MAPK/ERK pathway is a critical factor due to its dual role in myocardial ischemia, acting as a protector and a mediator of damage. The activation of ERK1/2 involves fibroblast proliferation, which is vital for survival during ischemic conditions. Its activation reduces cardiomyocyte apoptosis and inhibits Rho‐kinase‐mediated damage, thus acting as a main pathway in myocardial conservation against ischemia (Zhang et al. 2024). Chen et al. (2017) investigated the cardioprotective impact of carvacrol (25, 50, and 100 mg/kg) against myocardial ischemia by activating the MAPK/ERK and Akt/eNOS pathways in Wistar rats. They reported that carvacrol improved SOD and CAT and reduced MDA levels.

Bradycardia, slower heart rates, often involves impaired TRPM7 function that results in decreased heart rate. TRPM7, a key ion channel‐kinase, keeps heart rate and cardiac automaticity by modulating pacemaker activity. The proper function of TRPM7 requires accurate calcium/magnesium signaling and HCN4 channel. Its association with cardiac fibrosis and cardiac failure makes it a potential target in the management of heart issues (Hu et al. 2021). However, carvacrol can downregulate TRPM7 to maintain heart health. Alves et al. (2016) verified that carvacrol (100 μM) inhibited TRPM7 channels and reduced input and output in human atrial myocytes. Thymol (10–100 mg/kg) has been shown to mitigate inflammation and promote skeletal muscle recovery in cardiotoxic mice (Cardoso et al. 2016). Yu et al. (2016) reported that thymol (3, 6 mg/kg) reduced OS, TGs, TC, and LDL, and improved HDL in hyperlipidemic rabbits. Moreover, thymol supplementation inhibited IL‐1β, IL‐6, TNF‐α, MCP‐1, and VCAM‐1. Thymol (7.5 mg/kg) reduced lipid peroxidation and TGs, modulated Ca^2+^, and improved SOD, GSH, GPx, CAT, MDH, and α‐KGDH in myocardial infarcted rats induced by β‐adrenergic and isoproterenol (Meeran et al. 2016).

Thymol (53.68 μM), along with thymoquinone and artemisinin, reduced SphK1 activity and showed cytotoxicity and anti‐proliferative effect on H1299 and A549 lung cancer cells (Shakeel et al. 2024). Thymol, when combined with radiation therapy, induced cytotoxic and anticancer effects in A549 and MRC‐5 lung cancer cells (dos Santos et al. 2021). Gholijani et al. (2016) studied the effects of thymol and carvacrol on LPS‐induced pulmonary inflammation and reported that combined therapy significantly decreased IL‐1β and TNF‐α levels and modulated the expression of NFAT, STAT3, AP‐1, and JNK. Carvacrol (20, 40, and 80 mg/kg) protected Wistar rats against bleomycin‐induced lung inflammation, improved SOD, CAT, GSH, and thiol, and reduced TGF‐β1 and TNF‐α (Pashmforosh et al. 2024). Eriten et al. (2024) reported that carvacrol (25, 50 mg/kg) removed mercuric chloride (HgCl2) lung toxicity via improved SOD, GSH, GPx, Bax, caspase‐3/6/9, Apaf1, and p53 activity, while inhibiting Beclin‐1, LC3A, LC3B, NLRP3, COX‐2, NF‐κB, TNF‐α, iNOS, IκB, IL‐1β, and IL‐6. Table 6 shows the cardio‐pulmonary protective role of thymol and carvacrol.

**TABLE 6:** Cardio‐pulmonary protective role of thymol and carvacrol.

	Compound	Dose	In vivo/in vitro	Mechanism	References
Cardio‐protection	Carvacrol	5, 10, 25 and 50 mg/kg	Wistar rats	**↓**Bad mRNA, **↑**Bcl‐xL mRNA	Sadeghzadeh et al. (2018)
	Carvacrol	25, 50, 75, 150 and 300 μg/rat	Wistar rats	**↓**salt appetite, alpha‐adrenergic	de Souza Polli et al. (2019)
	Carvacrol	0.03–3 μM	In vitro/Sprague–Dawley rats	**↓**NOX 1, MAPK, proliferation	Lee et al. (2015)
	Carvacrol	5, 10 mg/kg	Sprague–Dawley rats	**↑**GSH, TAS, **↓**lipid peroxidation	Cetik et al. (2015)
	Thymol	5–25 μg/mL	THP‐1 monocytes	**↓**TNF‐*α*, IL‐6, **↑**IL‐10	Ocaña and Reglero (2012)
	Thymol	7.5 mg/kg	Wistar rats	**↓**IL‐1β, TNF‐α, IL‐6	Meeran et al. (2015)
Pulmonary protection	Thymol	50 mg/kg	BALB/c mice	**↓**TNF‐α, IL‐1β, MDA, NF‐κB, **↑**Nrf2, HO‐1	Yao et al. (2018)
	Thymol	100 mg/kg	BALB/c mice	**↓**TNF‐α, IL‐6, MDA, NF‐κB	Wan et al. (2018)
	Thymol + carvacrol	200 μM	Beas‐2B, A549, H292 cells	**↓**IL‐25, IL‐33, TLR4, TLR2, **↑**SHIP1, SOCS1	Khosravi and Erle (2016)
	Carvacrol NPs	240 μg/mL	Wistar rats	**↓**MDA, OS, inhalation injury	Carvalho et al. (2020)
	Carvacrol NPs	0–200 μg/mL	A549 cells	**↓**MAPK p38, ERK, VEGF, CD31, COX‐2	Khan et al. (2019)

## 14 Thymol and Carvacrol in Nervous System Disorders

The nervous system (NS) is a highly complex network accountable for receiving, processing, and responding to sensory information. Numerous neurodegenerative disorders like dementia, epilepsy, Alzheimer's disease, and depression disturb millions of individuals worldwide (Xu et al. 2022). Thymol and carvacrol have been found effective in mitigating CNS problems. It has been reported that carvacrol (20, 80 mg/kg) effectively reduces brain inflammation induced by paraquat in rats. Furthermore, carvacrol improved thiol, CAT, and SOD in animals (Khosravi et al. 2024). Durmus et al. (2024) demonstrated the neuroprotective effect of carvacrol against acrylamide‐induced brain toxicity in rats. They supplemented animals with 50 mg/kg/BW carvacrol and found that MDA, Nrf2, and NF‐κB levels reduced, whereas GSH and NR4A2 improved in rats. Nrf2 is a key player in oxidative stress, interacts with Keap1 to regulate cellular response against oxidative damage. Its abnormal function could lead to increased ROS production and oxidative damage to vital organs such as brain. Additionally, NR4A2 also referred as NURR1is a critical nuclear receptor transcription factor vital for the development and survival of midbrain dopaminergic neurons. Its connection with immune response and inflammation makes it a significant factor in brain and neurodegenerative disorders (Ruiz‐Sánchez et al. 2025). Forqani et al. (2023) demonstrated that carvacrol (15 or 30 mg/kg) enhanced memory and reduced oxidative damage to brain tissue in aged male rats by increasing thiol levels and decreasing MDA levels. Carvacrol (100, 200 mg/kg) alleviated TBI‐induced brain inflammation in rats by modulating the caspase‐3/NFκB p65 pathway, and reduced IL‐1β, TNF‐α, and IL‐6 (Abbasloo et al. 2023).

**FIGURE 6:**
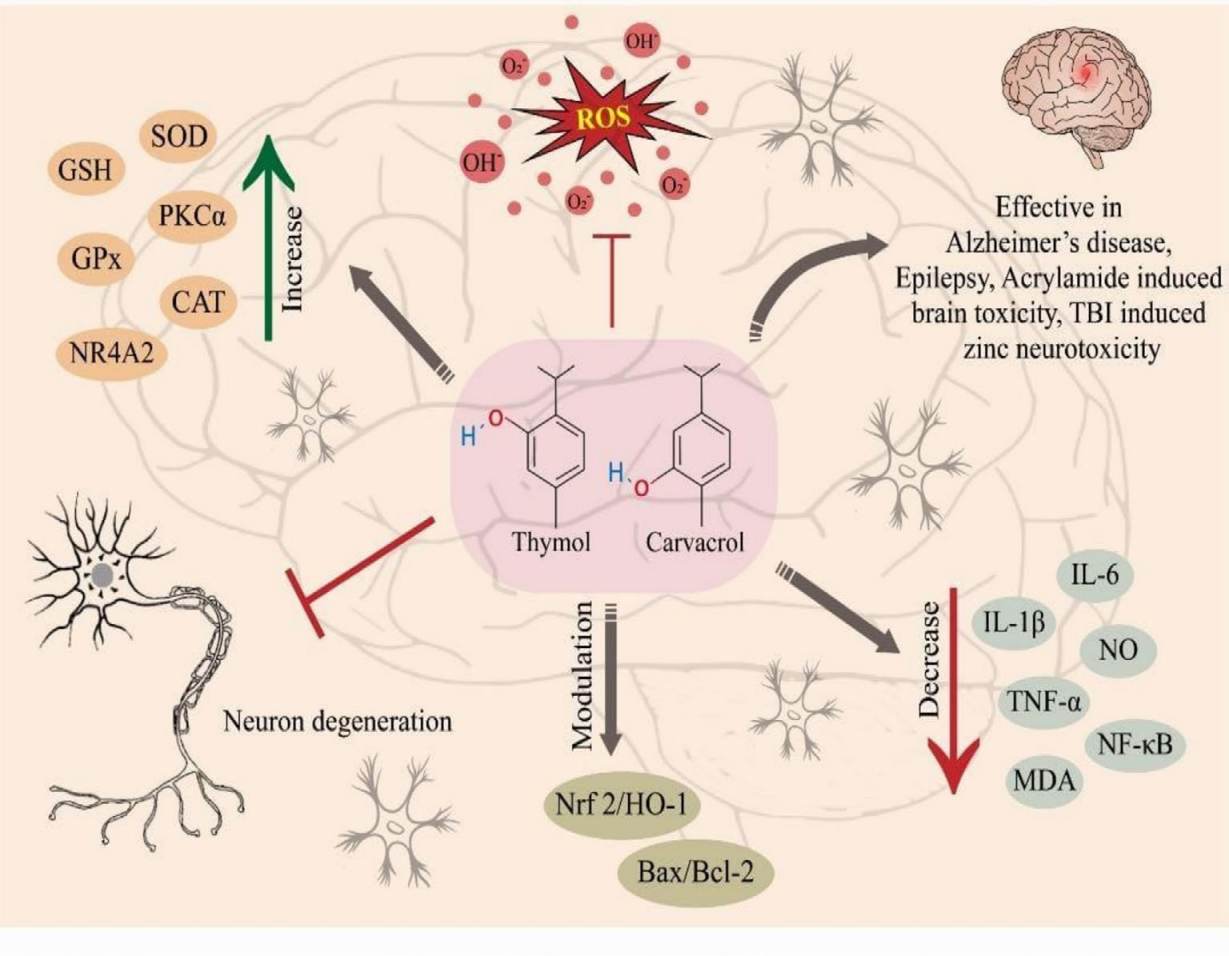
Protective impact thymol and carvacrol in nervous disorders via modulating Nrf2/HO‐1 and Bax/Bcl‐2 expression, reducing IL‐1β, TNF‐α, NO, IL‐6, MDA, and NF‐κβ, inhibiting ROS production, and increasing SOD, GPx, CAT, PKCa, and NR4A2.

Previously, Lee et al. (2022) proved that carvacrol (50 mg/kg) inhibited TBI‐induced zinc neurotoxicity and TRPM7 expression in rats' brain injury. Hakimi et al. (2020) reported that 25, 50, or 100 mg/kg carvacrol can inhibit MDA, IL‐6, and NO and improve SOD, CAT, and thiol in LPS‐induced OS and inflammation in rats' brains. Previously, carvacrol (50 mg/kg) has been shown to be efficacious against PCZ‐induced brain damage, with improvements in GPx, SOD, and GSH in rats (Elhady et al. 2019). Amyloid‐beta (Aβ) has been associated with nerve damage and memory deterioration. However, thymol and carvacrol can protect CA1 pyramidal neurons from Alzheimer's by improving PKC activity and increasing PKCα expression (Azizi et al. 2022). The link between Nrf2 and HO‐1 has been widely discussed in various studies. Nrf2 coordinates with HO‐1 in neuroprotection. Moreover, P‐Ser9 GSK3β is also crucial in neuroprotection because it is responsible for neurogenesis and inhibits tau hyperphosphorylation (Lai et al. 2023). Thymol (0, 40 mg/kg) has been found to be effective in reducing brain insulin resistance, modulating Nrf2/HO‐1 signaling, and enhancing P‐Ser9 GSK3β expression in HFD‐induced cerebral impairments in C57BL/6 J mice (Fang et al. 2017). Aydın et al. (2017) proved that thymol (400 mg/L) reduced cell viability in rat neurons, 200 and 400 mg/L thymol inhibited N2a cells, and 10, 25, and 50 mg/L enhanced antioxidant activity in rat neurons. The protective impact of thymol and carvacrol against CNS disorders is demonstrated in Figure 6.

## 15 Antimicrobial Activities

The antimicrobial activity of thymol and carvacrol has been reported in several studies. Miranda‐Cadena et al. (2021) reported that thymol inhibited various *Candida* spp. with a minimum inhibitory concentration of 161.3 mg/L. Thymol also proved effective in reducing several bacterial strains. Peter et al. (2024) and Chen et al. (2021) reported thymol's antibacterial activity against *S. aureus* , *E. coli* , and *S. epidermidis* , with MIC values (0.25–3.3 μg/mL). Carvacrol showed an antifungal effect against *A. flavus* , *C. glabrata* , and *M. furfur* (Duan et al. 2024). Table 7 illuminates the antimicrobial potential of both monoterpenes against a wide range of microorganisms.

**TABLE 7:** Antimicrobial activities of thymol and carvacrol.

	Microorganism	Strains	MIC range	References
Thymol	Fungi	*C. albicans* , *C. tropicalis*	25, 50 and 25 μg/mL	Jafri and Ahmad (2020)
	Fungi	*C. albicans*	247 μg/mL	Niu et al. (2020)
	Fungi	*C. dubliniensis C. guilliermondii*, *C. orthopsilosis*, and *C. tropicalis*	161.3 mg/L	Miranda‐Cadena et al. (2021)
	Bacteria	*E. faecalis* , *E. coli and L. monocytogenes*	39–1250 μg mL^−1^	Giacomin et al. (2024)
	Bacteria	*S. aureus* , *E. coli*	0.50 and 0.25 mg/mL	Chen et al. (2021)
	Bacteria	*S. aureus* , *S. epidermidis*	1.25–3.3 μg/mL	Peter et al. (2024)
Carvacrol	Bacteria	*S. aureus* , *P. aeruginosa* , *E. coli*	81, 128, 5 μg/mL	da Silva et al. (2023)
	Bacteria	*S. aureus* , *E. coli*	0.22, 12.5 mg/mL	Wu, Xia, et al. (2023)
	Bacteria	*S. aureus* , *E. coli*	125, and 500 μg/mL	Liu, Wang, et al. (2022)
	Bacteria	*E. coli*	250 μg/mL	Asadi et al. (2023)
	Fungi	*C. albicans* , *C. glabrata* , *C. tropicalis* , *C. krusei*	780–1560 μg/mL	Vitali et al. (2021)
	Fungi	*A. flavus*	0.60 μL/mL	Duan et al. (2024)
	Fungi	*M. furfur*	32 μg/mL	Vinciguerra et al. (2019)
	Fungi	*B. cinerea*	140 μL/L	Zhang et al. (2019)

## 16 Clinical Trails

Clinical trials on thymol and carvacrol have proved their health‐promoting and disease‐preventive role in humans. In a clinical trial, 30 diabetic participants were divided into 4 groups and treated with laser therapy, thymol (25 mg/kg), and a combined therapy of thymol and laser for 30 days. Moreover, thymol gel oil extract (0.5%) was also applied to evaluate its impact on dermatitis in the feet of the diabetic group. The findings revealed that combined therapy significantly reduced MDA, IL‐1α, IL‐1β, and TNF‐α. Additionally, the combination also lowered LDL, TC, and HbA1c. However, thymol gel (0.5%) showed a non‐significant impact (Martirosyan et al. 2022). In a double‐blind clinical trial, chlorhexidine‐fluoride varnish (CHX‐F) or chlorhexidine‐thymol varnish (CHX‐T, Cervitec Plus) was assessed as a control against *Streptococcus mutans* for 24 weeks on 57 healthy school children. The results indicated that the CHX‐F and CHX‐T varnishes did not have a substantial difference in terms of *Streptococcus mutans* inhibition following four applications (Lipták et al. 2016). A randomized phase I clinical study was conducted to determine the safety of carvacrol in 40 healthy male and female participants (aged 20–40). The subjects were divided into two groups of 20 each in doses of 1 and 2 mg/kg/day. These findings demonstrated that the levels of calcium, ESR, MCV, and Hb had decreased, whereas the creatine phosphokinase (CPK) levels were elevated in the carvacrol (1 mg/kg/day) group. Moreover, the 2 mg/kg/day carvacrol group showed decreased HDL, total bilirubin, iron, and RBC count, and increased FEV1. However, no clinical toxicity was detected in the subjects, and all of the parameters were in normal range (Ghorani, Alavinezhad, Rajabi, and Boskabady 2021). A randomized clinical trial was conducted to evaluate carvacrol effects on respiratory symptoms in asthmatic patients, involving 33 subjects, comprising a normal and carvacrol group (1.2 mg/kg/day). The study concluded that the carvacrol capsules administered 3 times/day over 2 months were found to reduce respiratory symptoms, pulmonary function tests (PFTs), and SOD, thiol, and CAT levels (Ghorani, Alavinezhad, Rajabi, and Boskabady 2021).

## 17 Safety and Toxicity Reports

The safety of the plants and bioactive compounds is crucial before their application, due to health risks and adverse effects. Besides, evidence‐based reports do not have adequate information regarding their safety profiles. Nevertheless, the European Food Safety Authority (EFSA) reported that thymol is considered safe at 1000 mg/kg body weight, but at high doses, thymol toxicity and adverse effects occur. Carvacrol is non‐toxic at low doses; however, a concentration higher than 2480 mg/kg body weight can cause toxicity (Ghorani, Alavinezhad, Rajabi, Mohammadpour, and Boskabady 2021). Abbas et al. (2017) have reported health‐related risks and toxicity of carvacrol and thymol. Table 8 explored the safety and toxicity reports of thymol and carvacrol.

**TABLE 8:** Safety and toxicity reports of Thymol and Carvacrol.

	Dose/concentration	Study type	Adverse impact	References
Thymol	150, 750 and 3750 μg/mL	In vitro	✔	Cohen et al. (2021)
	130, 260 and 360 μM	In vitro	✔	Hikiba et al. (2005)
	25, 50, 75, and 100 μg/ml	In vitro	✔	Buyukleyla and Rencuzogullari (2009)
	20, 40 and 80 μg/mL	In vitro	✔	Kusakabe et al. (2002)
	16–250 μM	In vitro	✘	Maisanaba et al. (2015)
	0–250 μM	In vitro	✘	Llana‐Ruiz‐Cabello et al. (2014)
	600 μM	In vitro	✘	Horvathova et al. (2007)
Carvacrol	1, 2 mg/kg/day	In vivo	✘	Ghorani, Alavinezhad, Rajabi, Mohammadpour, and Boskabady (2021)
	81, 256, 810 mg/kg	In vivo	✘	Llana‐Ruiz‐Cabello et al. (2016)
	25 μg/kg	In vivo	✘	Ribeiro et al. (2019)
	0.01–1.0 μL/plate	In vitro	✘	Ipek et al. (2005)
	5 μL/mL	In vitro	✘	Ipek et al. (2003)
	10, 25, 50, 75, 100, 150, 200 μg/mL	In vitro	✘	Türkez and Aydın (2016)
	30, 60 mg/kg	Ex vivo	✘	Slamenova et al. (2011)
	100 and 500 mg/kg	In vivo	✔	Stojanović et al. (2018)

## 18 Applications and Limitations

The applications of thymol and carvacrol are common in medicine, veterinary science, food, pharmaceutics, and cosmetics markets. The antimicrobial effect makes both thymol and carvacrol effective in the field of oral hygiene and health (Rezaeian et al. 2019; Botelho et al. 2007). Listerine antiseptic mouthwash and Cervitec Plus protective varnish are two commercial products comprising thymol used for oral health and to manage bacterial plaque, cavities, and gingivitis (Kokoska et al. 2019). Furthermore, animal‐based studies have also proven the beneficial role of thymol and carvacrol. Regarding this, André et al. (2017) stated that thymol exhibited anthelmintic effects against the sheep gastrointestinal nematodes. Arafa et al. (2020) stated that thymol is effective against coccidiosis in pigeons caused by apicomplexan protozoan.

The antimicrobial properties of thymol and carvacrol make them suitable candidates for natural preservatives in the food sector. However, their pungent aroma and taste overwhelmed the food's original flavor and aroma. Therefore, the combination of these two compounds with other ingredients can reduce this drawback and also enhance their antimicrobial potential. Thymol and carvacrol (0.5–2.0 mM) with NaCl (1%–15%) at 22°C inhibited *S. aureus*, *E. coli* , and *L. monocytogenes* growth. The findings showed that thymol or carvacrol (2.0 mM) and NaCl (≥ 3%) completely inactivated bacterial population (Kim et al. 2020). The solubility of thymol is another limitation to its application, as thymol is hydrophobic, which inhibits its dispersion in aqueous medium. Considering this, Li et al. (2017) developed hydrophilic–lipophilic surfactant‐based thymol nanoemulsions and tested their preservative activity on lettuce and blueberries. To enhance the shelf life of fruits sulfur dioxide has been mostly used; however, the safety and regulatory issues linked with such substances have increased the need to adopt safe and more effective strategies. Thymol with natural components have proved more efficient to enhance the shelf life and quality of fruits. Longan fruit was treated with thymol (5%) and 2% chitosan in combination with 5% ascorbic acid, citric acid, oxalic acid and modified atmosphere packaging. The findings revealed that the treatment improved fruit quality and enhanced shelf life for 56 days by preventing pericarp browning (Khan et al. 2020). Thymol and carvacrol as biodegradable packaging have been studied in multiple studies. Li et al. (2020) investigated the antimicrobial properties of thymol‐gelatin and soy lecithin nanoemulsion films against both gram‐positive and gram‐negative bacteria, reporting an improved shelf life of foods. Tas et al. (2019) developed a chitosan and halloysite nanotubes‐based carvacrol‐loaded coating film and checked it against *A. hydrophila* on chicken meat. The results demonstrated that coatings inhibited 85% viability of *A. hydrophila* .

**FIGURE 7:**
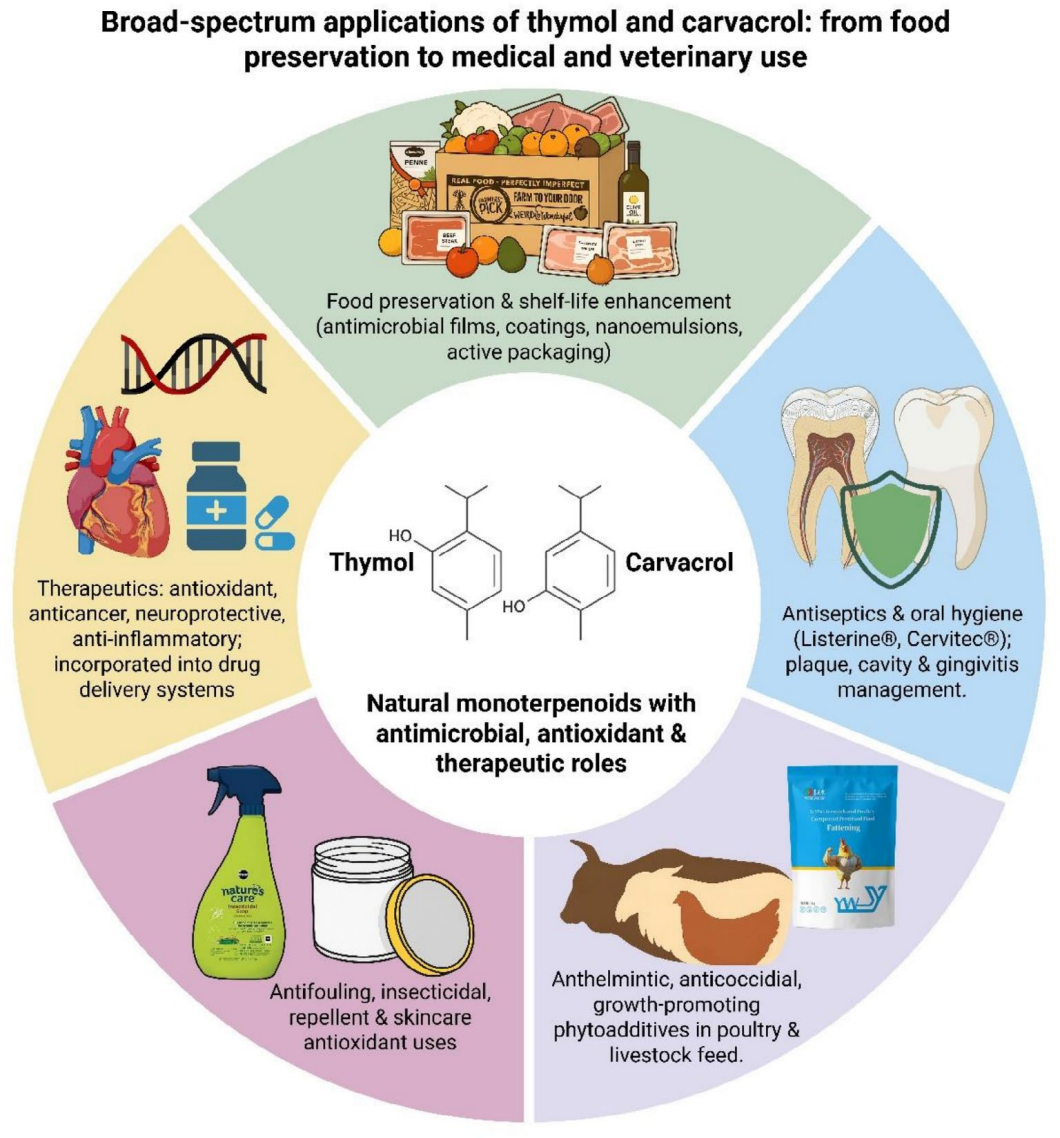
Applications of thymol and carvacrol in medical and pharmaceutical science, veterinary science, food sector, and dentistry.

Phyto additives, such as thymol and carvacrol, with strong antimicrobial, antiparasitic, and antioxidant activities, have garnered attention in research. However, their suitable dose and mechanism of action need to be explored. The addition of thymol in animal feed to improve their growth is another valuable application. Placha et al. (2019) applied 0.5 g/kg of thyme oil to broiler chicken feed and concluded that thyme oil reduced MDA and improved IgA and SOD. Moreover, thymol was detected in plasma, the duodenal wall, and breast muscle, thereby enhancing the antioxidant system and promoting overall growth. Ocel'ova et al. (2016) proposed that thymol content was abundant in the liver and kidney rather than in chicken muscles. Haselmeyer et al. (2015) fed chickens with thyme leaves and flowers for 35 days and found thymol in the intestine, plasma, and liver. Thyme oil (200, 300, and 400 ppm) with 35%–40% thymol for 35 days reduced TGs and inhibited *E. coli* in quail chicks, compared with flavophospholipol (100 ppm) (Dehghani et al. 2019). Thymol and carvacrol possess insecticidal and repellent attributes (Tabari et al. 2017), antifouling properties (Pérez et al. 2015), and act as agrochemical agents (Cheng et al. 2020). Taken together, thymol and carvacrol have various applications in different fields showcasing their multisectoral use. However, their pungent aroma in food industries, bioavailability in pharmaceutics, and lack of clinical studies are major limitations. Although methods have been developed to overcome these challenges, robust researches are still required to validate their final applications. The applications of thymol and carvacrol in various fields are displayed in Figure 7.

## 19 Conclusion and Future Perspectives

*Thymus serpyllum* , which contains thymol and carvacrol as major monoterpenoid bioactive compounds, is a viable approach to preventing disease. Thymol is a colorless hydrophobic substance that dissolves in alcohols. It is recognized as an antioxidant, anti‐inflammatory, anti‐microbial, and anticancer agent. Carvacrol is an isomer of thymol with antidiabetes, anticancer, antihypertensive, immunomodulatory, and hepatorenal protective potential. Although promising in their pharmacological properties and uses, the two compounds have limited clinical trials, lacking in extraction and stability technologies and methods to promote the bioavailability. Research in the future should focus on effective clinical studies to determine safety, efficacy, optimum dosage of these compounds, and bioavailability. Combining thymol and carvacrol with other phytochemicals, plant extracts, or conventional drugs may enhance synergistic therapeutic effects. Moreover, future research should also emphasize molecular‐level investigations to clarify their precise mechanisms of action in inflammation, oxidative stress, and disease‐specific pathways. Advanced omics approaches, including genomics, proteomics, and metabolomics, can help identify novel therapeutic targets and biomarkers of response. Additionally, evaluating their effects on gut microbiota modulation may reveal new roles in metabolic and immune health. Sustainable cultivation practices for *T. serpyllum* , standardization of extracts, and quality control protocols should also be prioritized. Finally, Novel delivery systems are needed to improve their usage in targeted therapy, functional foods, and integrative medicine.

## Author Contributions

**Shehnshah Zafar** contributed to investigation, data curation, and writing – original draft. **Muhammad Tauseef Sultan** was involved in methodology, formal analysis, and writing – review and editing. **Ahmad Mujtaba Noman** contributed to validation, visualization, and writing – review and editing. **Hassan Raza** provided supervision, resources, and contributed to writing – review and editing. **Aimen Mazhar** was responsible for methodology, writing – original draft, and data curation. **Farhang Hameed Awlqadr** contributed to writing – original draft, writing – review and editing, conceptualization, resources, and supervision. **Duaa Tariq** contributed to writing – review and editing, validation, and visualization. **Khaled Arab** was responsible for writing – review and editing, methodology, validation, and visualization. **Muhammed Noori Saeed:** software, formal analysis, and data curation.

## Funding

The authors have nothing to report.

## Conflicts of Interest

The authors declare no conflicts of interest.

## Data Availability

The data that support the findings of this study are available from the corresponding author upon reasonable request.
